# Ensemble averaging: What can we learn from skewed feature distributions?

**DOI:** 10.1167/jov.23.1.5

**Published:** 2023-01-05

**Authors:** Aleksei U. Iakovlev, Igor S. Utochkin

**Affiliations:** 1 University of Iceland, Reykjavík, Iceland; 1 HSE University, Moscow, Russia; 1 Institute for Mind and Biology, University of Chicago, Chicago, IL, USA

**Keywords:** ensemble perception, robust averaging, population coding, sampling, feature distribution, pooling

## Abstract

Many studies have shown that observers can accurately estimate the average feature of a group of objects. However, the way the visual system relies on the information from each individual item is still under debate. Some models suggest some or all items sampled and averaged arithmetically. Another strategy implies “robust averaging,” when middle elements gain greater weight than outliers. One version of a robust averaging model was recently suggested by Teng et al. (2021), who studied motion direction averaging in skewed feature distributions and found systematic biases toward their modes. They interpreted these biases as evidence for robust averaging and suggested a probabilistic weighting model based on minimization of the virtual loss function. In four experiments, we replicated systematic skew-related biases in another feature domain, namely, orientation averaging. Importantly, we show that the magnitude of the bias is not determined by the locations of the mean or mode alone, but is substantially defined by the shape of the whole feature distribution. We test a model that accounts for such distribution-dependent biases and robust averaging in a biologically plausible way. The model is based on well-established mechanisms of spatial pooling and population encoding of local features by neurons with large receptive fields. Both the loss functions model and the population coding model with a winner-take-all decoding rule accurately predicted the observed patterns, suggesting that the pooled population response model can be considered a neural implementation of the computational algorithms of information sampling and robust averaging in ensemble perception.

## Introduction

In the real world, we are often surrounded by big groups of similar objects that we can perceive in a generalized way, or as an ensemble. For instance, one may encounter a tree with many leaves varying from green to yellow and red while wandering around a park in autumn. Because there are thousands of leaves on just a single tree, deep perceptual processing of each at the same time is beyond any reasonable capacity for the visual system. Ensemble perception offers us a strategy of describing all things simultaneously with a set of summary statistics, such as the average color or size of all the leaves or the variance of colors or sizes. Research has demonstrated that observers can judge summary statistics accurately and with quite a brief presentation (Chong & Treisman, 2003; Khayat & Hochstein, 2018; Whiting & Oriet, 2011), whereas their conscious access to the features of individual items is severely limited (Ariely, 2001; Parkes, Lund, Angelucci, Solomon, & Morgan, 2001). It is also shown that the perception of summary statistics is ubiquitous across feature domains from basic dimensions, such as orientation (Dakin, 2001; Solomon, 2010), size (Ariely, 2001; Chong & Treisman, 2003), motion speed and direction (Watamaniuk & Duchon, 1992; Watamaniuk, Sekuler, & Williams, 1989), brightness (Bauer, 2009; Khayat & Hochstein, 2018), or color (Maule & Franklin, 2015), to more complex features such as facial expression or identity (de Fockert & Wolfenstein, 2009; Haberman & Whitney, 2007), or even more abstract semantic features such as animacy (Leib, Kosovicheva, & Whitney, 2016), object category (Khayat & Hochstein, 2019), or economic value (Yamanashi Leib, Chang, Xia, Peng, & Whitney, 2020).

The computational mechanism of ensemble perception is under discussion. One of the crucial questions is whether the visual system indeed summarizes all of the items equally with no capacity limit or whether it makes an approximation based on only a few items. Some theories suggest that ensemble processing is a coarse mode of processing applied virtually to the entire set simultaneously (Ariely, 2001, 2008; Baek & Chong, 2020a, 2020b; Treisman, 2006). Other theories portray ensemble perception as sampling with quite low efficiency in terms of the number of items that carry useful information. Although the accuracy of summary statistical judgments fairly overcomes that of judgments of individual features, numerous computational models claim that this level of accuracy could be effectively accomplished if observers effectively sampled a small fraction of items (Allik, Toom, Raidvee, Averin, & Kreegipuu, 2013; Dakin, 2001; Gorea, Belkoura, & Solomon, 2014; Im & Halberda, 2013; Marchant, Simons, & de Fockert, 2013; Maule & Franklin, 2016; Myczek & Simons, 2008; Solomon, 2010; Solomon, Morgan, & Chubb, 2011). In most such theories, an otherwise-ideal observer randomly picks a few items corrupted by the early noise and takes the arithmetic mean of this sample, and finally the late noise is applied. As summarized by Whitney and Yamanashi Leib (2018), the ideal observer would perform at a level shown on the real data if the effective sample size grows approximately as a square root of presented set size (see also Dakin, 2001; Solomon et al., 2011).

Although the standard versions of the sampling models rely on random sampling and arithmetic averaging, other theories suggest more complex strategies of averaging. Some of these strategies limit randomness (such as relative amplification of more salient items and giving them more weight) (Goldenberg, Weisz, Sweeny, Cikara, & Gross, 2021; Iakovlev & Utochkin, 2021; Kanaya, Hayashi, & Whitney, 2018). Other strategies, in addition, do not even imply arithmetic averaging. One important example is so-called robust averaging (de Gardelle & Summerfield, 2011; Li et al., 2017) that gives more weight to items that are close to the mean and less weight to items far from the mean. In this class of models, the visual system scales evidence obtained from each sampled element as a function of its distance from the reference against which the average should be compared (e.g. a boundary between different color categories in a color averaging task, as in de Gardelle & Summerfield, 2011; Li et al., 2017; or a single item of a particular orientation in an orientation averaging task, de Gardelle & Summerfield, 2011; Li, Castañón, Solomon, Vandormael, & Summerfield, 2017). In a model by de Gardelle and Summerfield (2011), the weighting function diminishes the weights of outlying elements by a log posterior-odds factor, reflecting the ratio between the probabilities of each response category given the feature value. In line with the idea of robust averaging, evidence was found that items that extremely deviate from the rest of the ensemble (outliers) are devalued in averaging (Epstein, Quilty-Dunn, Mandelbaum, & Emmanouil, 2020; Haberman & Whitney, 2010). Although robust averaging in general is considered a suboptimal strategy of information integration, as it gives unequal weights to equally reliable sources of information, Li et al. (2017) showed that it gives an advantage when decisions are subject to the strong late noise.

An important diagnostic case for understanding how items are sampled and weighed in ensemble perception was recently suggested by Teng, Li, and Zhang (2021). They tested how observers average directions of multiple moving dots in skewed distributions. Whereas symmetrical distributions (such as Gaussian or uniform distributions that are usually tested in ensemble experiments) usually have equal deviation from the mean to both sides, in skewed distributions these deviations are different. Since the mode of a skewed distribution is shifted relative to the mean, smaller deviations prevail on one side (the side of the mode) and bigger deviations prevail on another side (the side of the skew). Using such a design, Teng et al. (2021) found a remarkable systematic bias of estimated average directions away from the real mean toward the mode. Moreover, this bias tended to grow as the distance between the mode and the mean increased. This finding strongly argues against random sampling with arithmetic averaging because this mechanism predicts that estimates of the mean should be nonsystematically erroneous across multiple trials, but converge on the correct answer on average, because arithmetic averaging works in accordance with the central limit theorem. This finding is rather consistent with a version of the robust averaging strategy that devalues highly deviant samples. In accordance with Teng et al. (2021), their findings were best explained by a computational model with random sampling and robust averaging. Computationally, the mechanism suggested by Teng et al. (2021) can be described as follows: 1) random items are sampled during a trial over space and time, 2) a probabilistic weighting function (the so-called virtual loss function) is applied to each point in the motion direction space to find which point yields the minimal expected loss given the sample distribution, and 3) the late noise is added to this estimate. The virtual loss function is a central internal component of the model that maps distances from each point of the feature space to each sampled item onto the Bayesian expected loss and a minimal point of this function is an approximate of the average. The three steps of this model (that we term a “loss function model”) are related to three parameters: effective sample size, the width of the loss function, and late noise. Two former parameters are critical to predict averaging. The best shape of the loss function was found to be an inverse Gaussian, and its standard deviation (SD) defined its width. When the loss function was wide enough, the model successfully predicted the bias.

Although the aforementioned models perform well to make strong quantitative predictions about observer's behavior in ensemble tasks, they mostly suggest computational abstractions of how the visual system represents ensemble information. Do the concepts such as effective set size or various loss functions correspond to underlying computational processes carried out by the visual system? This question can be viewed from the perspective of imaginable mechanisms of neural encoding and decoding, their demands, required computational complexity and limitations. From that point of view, at least some of the concepts from these models can be questioned. For example, the implementation of any evidence-weighting function requires the visual system to encode a lot of relational information about each effectively sampled item for even more comparisons with the space of potential ensemble estimates. Second, weighting individual samples based on the posterior odds or the loss function implies that the whole distribution and its crucial parameters should be available before assigning different weights to individuals. This process leads to a somewhat circular mechanism: One gives less weight to outliers in mean estimation, but one should know where the mean (or the mode) is to know which items should be considered outliers. This circularity is partially bypassed if the observers judge the average relative to a reference (as in Gardelle & Summerfield, 2011; Li et al., 2017), so that each item is estimated in relation to this reference rather than ensemble mean itself. However, it is more problematic in absolute judgments, such as in Teng et al. (2021) adjustment task. Having said that, circularity in outlier discount is not a completely impossible thing. For example, Epstein et al. (2020) demonstrated that outlier discount takes time to develop suggesting that this might be an iterative process.

Given these complications that the discussed computational models as formal mathematical descriptions can encounter, we suggest that models based on neurally plausible mechanisms of visual processing can give additional insights into the nature of the observed behavioral patterns in the ensemble tasks. In the present study, we test a model that derives ensemble perception from the basic properties of the feedforward stream of information processing in the visual system. It is based on two very common and ubiquitous structural and functional properties of the visual and other sensory and motor systems: population coding (Georgopoulos, Schwartz, & Kettner, 1986; Pouget, Dayan, & Zemel, 2000) and hierarchically increasing receptive fields (RFs). The general idea of this model is that neural representations of individual items are first represented locally by neural populations with small RF, like those of V1, and then are pooled by neural populations with large RFs. The response of the pooling population can be read out as ensemble summary statistics. The outline of an idea that a joint population response to a set of items can be a biologically plausible mechanism of ensemble representation can be found in many authors (Baek & Chong, 2020a; Chong & Treisman, 2003; Haberman & Whitney, 2012; Hochstein, Pavlovskaya, Bonneh, & Soroker, 2018) and is supported by direct neurophysiological evidence (Treue, Hol, & Rauber, 2000). There are also computational implementations of this idea. For example, Brezis, Bronfman, and Usher (2017) presented a model that explains rapid averaging of multiple symbolic numbers. In this model, each number is encoded by a noisy population of neurons tuned to certain numeric magnitudes and then responses from all populations are pooled by a decoding layer of number-selective neurons with parabolic-shaped tuning curves. The peak of the pooled response is decoded as the mean. Dakin, Mareschal, and Bex (2005) and Webb, Ledgeway, and McGraw (2007, 2010) suggested population code models of motion and orientation integration, although their main focus was on distinguishing between different decoding mechanisms that would better account for behavioral data. In their recent work, Utochkin, Choi, and Chong (2022), developed a general population coding model of ensemble perception. They shift the main focus to pooling itself as a key part of ensemble encoding process and conceptualize the pooled population response as the neural representation of ensemble. The pooled population response has a lot of properties that are isomorphous to the feature distribution of a presented set that make it possible to read out various statistical information: not only central tendency (by peak location, although other decoding rules are also possible: Georgopoulos et al., 1986; Jazayeri & Movshon, 2006; Ma, Beck, Latham, & Pouget, 2006), but also variance (the width of the population response distribution) and even the shape of the distribution (e.g., whether this distribution has a single peak or several peaks). Computational simulations based on this model successfully capture ensemble perception patterns across statistical summaries, feature domains, and paradigms reported in the literature (Utochkin et al., 2022).

**Figure 1.**
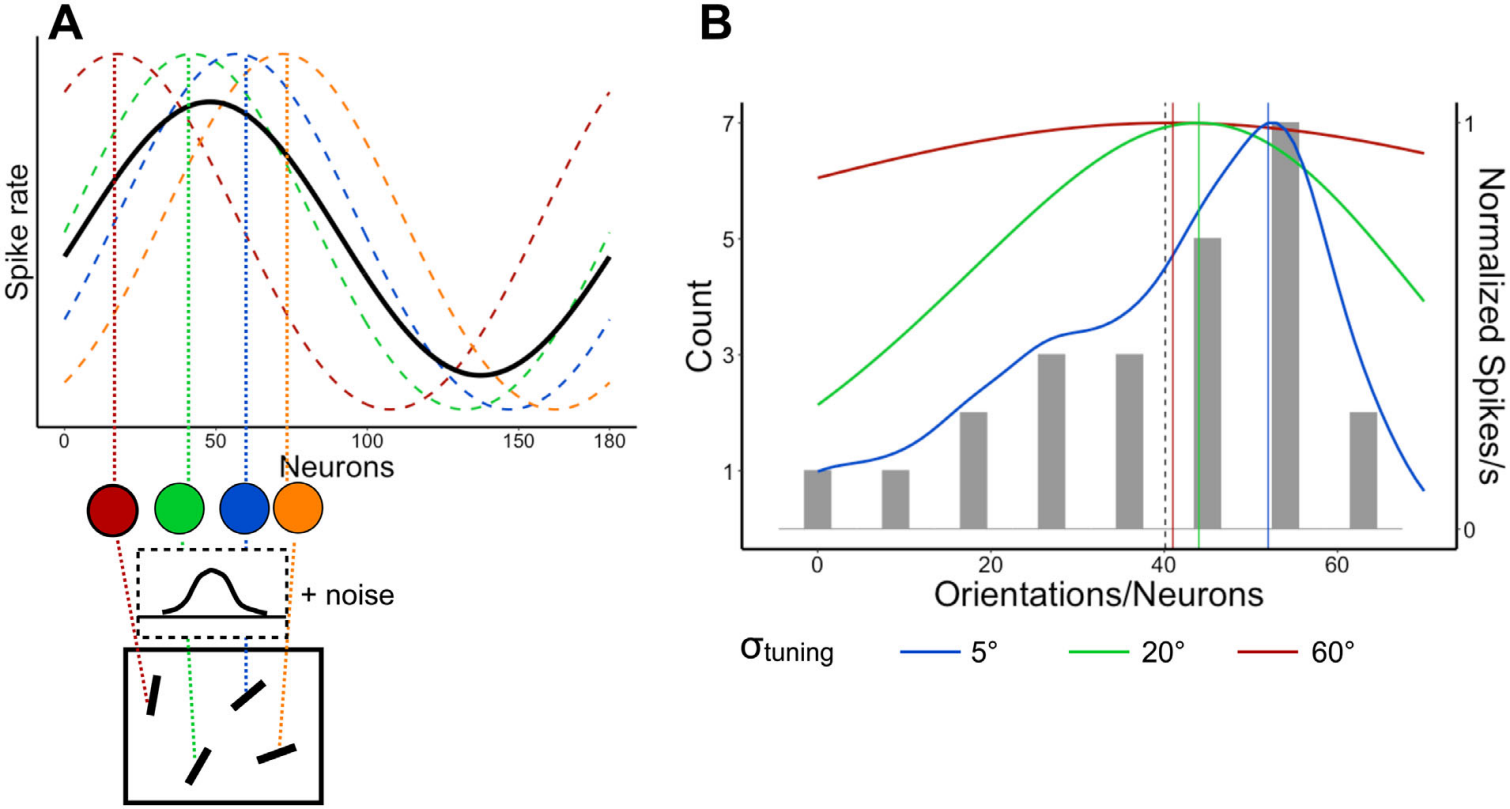
A population coding model of ensemble perception. (A) The general architecture of the model. Here, an example set of few oriented lines (mean orientation 50°) is transformed into four individual orientation values (color circles) after corruption by a Gaussian early noise. Each value is represented as a component population response (dashed color distribution lines). The component responses are pooled by averaging to produce an ensemble representation (black solid line) with a peak location decoded as the average. (B) A histogram of an example stimulus orientation distribution: The count axis showing the number of items per feature value) with three shapes of hypothetical population responses to this distribution depending on the width of the tuning curve. The narrower the tunings, the more its peak (shown by a solid color line in each case) shifts away from the real mean (dashed vertical line) toward the mode. For illustration purposes, the population response curves are normalized by their peak activation.

Here, we propose that the pooling and population coding model suggests a parsimonious and a biologically plausible explanation of robust averaging and outlier discount in general and the perception of skewed distributions in particular. We first explain the basic architecture of the model and then specify our predictions about the skewed distributions. The architecture of the model is shown for a simple case of the four-item set in Figure 1A. Each item is first represented as an individual sample corrupted by some amount of Gaussian early noise (this is a proxy for representing items in RFs with small RFs). The early noise (σ_early_) is one of the model's parameters. Each noisy item is then translated to a population response of a set of neurons ordered by their feature preferences at a top, pooling layer with a large RF. The population response is obtained by applying a normal (for linear feature dimensions) or a wrapped normal (for circular or semicircular spaces, such as orientation or motion direction) probability density function peaked at a neuron preferring a transmitted feature (given the noise) and a SD corresponding with the width of a tuning curve. When several such stimuli are presented, the pooled response is the average of population responses to individual stimuli or the normalized sum of the individual responses. It can be seen from Figure 1A that, when population responses to the individual features overlap, the resulting average population response will accumulate more signals in the middle than at the edges, so the middle of the range has more representation than extremes (which is reminiscent of the main point of robust averaging). The amount of the overlap is defined externally by the features physically presented and internally defined by the width of the tuning curve. Therefore, the SD of the Gaussian tuning curve (σ_tuning_) is a crucial parameter of the model. Finally, the peak of the pooled population response can be used to decode the best representative feature (which should be approximately the mean for a symmetrical feature distribution).

In particular, the population coding model predicts biases in the estimated averages when the feature distribution is skewed, such as those observed by Teng et al. (2021). Because the model is about signal accumulation from the feature distribution, it predicts the gravity of the peak response toward the mode. However, the magnitude of that gravity depends on the σ_tuning_. Figure 1B shows how the peak of a pooled response changes its location as a function of σ_tuning_. If a tuning curve is narrow, then the whole shape of the population response approaches the actual feature distribution and, hence, the peak gets closer to the mode. When the tuning curve gets wider, the peak shifts closer to the middle of the distribution, or the mean. It occurs because neurons, which preferentially respond to stimuli from the longer tail of a skewed distribution, gain more activation from prevailing features owing to the widespread tuning curve, and this can eventually shift the population peak away from the mode.

This study aims to test whether the pooling and population coding model can accurately account for averaging skewed distributions. Noteworthy, because the skewed distributions are shown sensitive to the way the individual items are sampled and weighed to accomplish the estimate of the average (Kim & Chong, 2020; Teng et al., 2021), testing their perception on its own can give valuable information about the nature of averaging. Based on that, we ran four experiments testing averaging of multiple orientations in skewed and nonskewed distributions to see whether findings by Teng et al. (2021) on motion averaging generalize to another feature domain. In Experiment 1, we tested experienced observers in a laboratory. Experiment 2 was an online replication of Experiment 1 with an extended group of naive observers. In Experiment 3, we tested another group of observers online with another set of skewed distributions. Finally, in Experiment 4, we tested whether the effect is replicated when observers adjust the mean orientation of a sample distribution using another distribution with the same or different skew as a probe. Anticipating the results, we replicated the systematic biases toward the mode in all of our experiments. We then applied the population coding model with three commonly implemented decoding rules (winner take all [WTA], vector averaging, and maximum likelihood) (Dakin et al., 2005; Webb, Ledgeway, & McGraw, 2007, 2010) to these data to test how accurate quantitative predictions this model provides. For comparison, we ran Teng et al's (2021) loss function model also developed to explain skewed distribution averaging to see whether the population response model can give the similar accuracy of predictions.

## Experiment 1

### Participants

Twelve experienced observers, five females, mean age 26.5 ± 6.8 years, including both authors, participated in the experiment. The participants other than the authors were naïve to the goal of the experiment. All had normal or corrected to normal vision and no neurological disorders. Before the experiment, the participants gave informed consent in accordance with the Declaration of Helsinki.

### Apparatus and stimuli

Stimuli were presented using PsychoPy 3 for Linux (Peirce et al., 2019) on a standard VGA monitor with a refresh frequency of 75 Hz and a 1,600 × 1,200-pixel spatial resolution on a homogeneous gray field. The viewing distance was about 50 cm. From that distance, one pixel subtended approximately 0.032° of visual angle. Stimuli were presented within a 400 × 400 pixels (approximately 12.8° × 12.8°) central square of a screen subdivided into a 5 × 5-cell imaginary grid. The central cell of the grid was used as the location of a fixation cross. The remaining 24 cells were used for positioning items from the sample set. Each cell contained a single item. Each item was located in the middle of a cell with a random jitter of 20 pixels along both axes.

**Figure 2.**
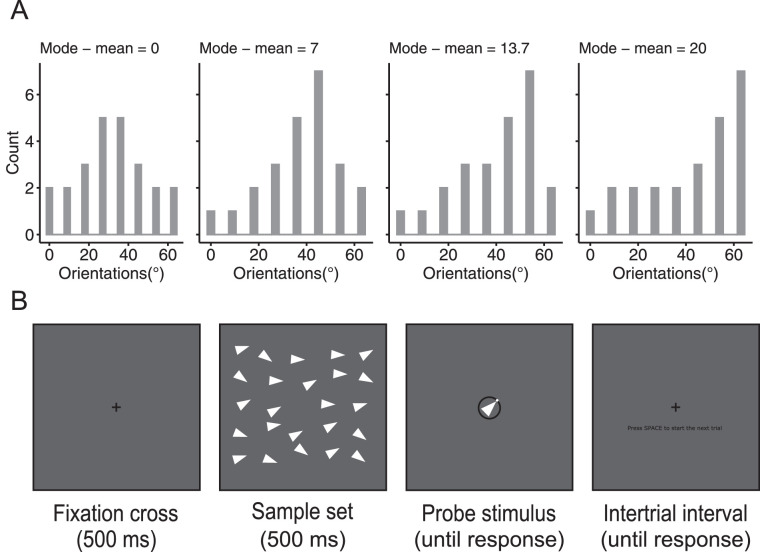
Stimuli and procedure of Experiments 1 and 2. (A) Orientation distributions from symmetric (mean–mode distance = 0) to most skewed (mode–mean distance = 20). Only left-skewed distributions are shown in this example, whereas the whole design included the right-skewed distributions as well. (B) The time course of a typical trial.

Sample sets consisted of 24 white isosceles triangles with various orientations. Triangles had 13 pixels width and 26 pixels height (0.42° × 0.84°). Base distributions of individual orientations covered the range from 0° to 63° with a step of 9°, but the proportion of each particular orientation differed as a function of the skewness condition. The distribution could be rotated by adding a random integer number from 1° to 360° to each orientation in each trial, which provided random assignment of the mean orientation. The skewness of the distribution was defined as the difference between the mode and the mean of orientation distribution and could take one of seven values: −20.0°, −13.7°, −7.0°, 0°, 7.0°, 13.7°, or 20.0°. Physically, these levels of skewness were accomplished using different proportions of triangles of different orientations. The histograms of the left-skewed distributions are shown in Figure 2A; right-skewed distributions were mirror symmetrical to left-skewed ones. The mean orientation itself was never presented in a set.

### Procedure

At the beginning of each trial, a fixation cross was presented for 500 ms followed by a sample set of triangles presented for another 500 ms (Figure 2B). Immediately after the set presentation, a probe stimulus was presented until response. The probe was a white triangle at fixation surrounded by a black ring with a white slider (white dot). The rotational position of the slider on the ring corresponded to the direction of the triangle's apex. Observers were instructed to adjust the orientation of the probe to match the mean orientation of a just seen set by clicking on or dragging the slider with the left button of a computer mouse. To confirm their final response, observers had to press a “space” button. During an intertrial interval, observers saw an instruction to press the space button to proceed to the next trial, which allowed the observers to progress with the experiment at a comfortable pace and take a rest whenever they wanted. Before the beginning of the experimental session, observers completed a block of seven practice trials to become familiar with stimuli and the procedure. After each practice trial, participants got feedback about their accuracy in the form of two triangles, one showing a correct mean orientation and another showing an adjusted orientation. During the experimental block of trials, no feedback was given.

### Design and data analysis

The experiment had a within-subject design with seven conditions of set skewness (−20.0°, −13.7°, −7.0°, 0°, 7.0°, 13.7°, and 20.0°). All conditions were presented in a random order within a single block of trials. Each participant completed 100 trials per condition (700 experimental trials in total).

In each trial, we calculated an adjustment error as the difference between a participant's response and the actual circular mean orientation of a feature distribution shown in this trial, using the following formula: Error = Response – Correct mean. The error could vary from −180° to 180°. A positive error value indicates a response more clockwise relative to the actual mean orientation, whereas a negative error indicates a more counterclockwise response. We then calculated the circular mean of an error distribution for each condition and each observer. This was used as a measure of *bias*, our dependent variable of interest. To analyze the change of the bias as a function of the feature distribution skewness, we used a linear mixed-effects model with the fixed effect of mode–mean distance and the random effect of participant's identity, slope, and intercept via lme4 package for R (Bates, Mächler, Bolker, & Walker, 2014). To calculate *p*-values for the regression we used Satterthwaite's (1946) method.

We also analyzed SDs of the error distributions for data exclusion if necessary. We excluded participants whose SDs of the error distribution exceeded 60°. This criterion was informed by a previous study where observers also adjusted the mean orientation of triangles (Utochkin & Brady, 2020). We also excluded individual trials if the error magnitude exceeded three SD of the entire error distribution for a given observer across all conditions. Based on these criteria we excluded 59 individual trials (approximately 0.7%) and none of the participants.

### Modeling

#### Population coding

We modeled ensemble encoding as a population response of neurons pooling noisy local signals about individual orientations presented in the stimulus. The model takes all presented orientations as an input: *S* ∈ {*S*_1_, *S*_2_, … *S*_n_}, where *S*_1...n_ are individual orientations in degrees. Each orientation from *S* is corrupted by Gaussian early noise in layer 1 of the model (σ_early_), so that the output of layer 1 is *S’* = *S + η*, where *η* are independent random samples from a normal distribution with a mean of 0 and a variance of σ^2^_early_. We set the σ_early_ = 4° as a fixed parameter of the model based on the average fits from the previous studies of orientation averaging (Dakin, 2001; Solomon et al., 2011). Each output number of layer 1 is a proxi of neural activity within a small V1-like RF responding to a single orientation falling onto this RF. Although the biologically plausible neural activity in each such RF is also a noisy population response of multiple neurons, we used the proxies for the sake of computational simplicity. We assume that this simplification is affordable by our model because it is focused on the shape of population responses at layer 2 of the model. Because the tuning curve of any neuron of interest can be presented directly as a function of the stimulus, there is no need to model the whole population response of intermediate neurons (which are layer 1 neurons in our case). Such a representation of the layer 1 output can also be interpreted as intermediate noisy decoding of individual orientations before this information is carried over to the pooling stage.

Layer 1 units representing each individual orientation are all connected to the same population of layer 2 neurons. Layer 2 applies to each noisy item conveyed by layer 1, a wrapped normal tuning curve (*f _WN_*) scaled to convert probability density units into spike frequency units, so that each item elicits a distribution of spikes in the whole layer 2 population in accordance with this tuning function:

(1) $$\begin{array}{c} a\left(\theta_{j};S{{}^{'}}_{i},\sigma_{tuning}\right)=M\times f_{WN}\left(\theta_{j};S{{}^{'}}_{i},\sigma_{tuning}\right),\; \end{array}$$

where θ*_j_* is an orientation preference of the *j*-th layer 2 neuron, Sʹ*_i_* is the noisy representation of layer 1 of the *i*-th item from the stimulus display as defined elsewhere in this article, σ_tuning_ is the width of the tuning curve of layer 2 neurons approximated with a wrapped normal distribution, and *M* = 60/max (*f _WN_* [θ*_j_* ; *S_i_* ʹ, σ_tuning_]), which converts probability densities into spike frequencies, so that a neuron at peak of its preference responds with 60 spikes/s. The σ_tuning_ was a free parameter of the model that we fit to the data.

The population responses to each individual item are summed and normalized (averaged) providing a resulting ensemble population response where each *j*-th layer 2’s neuron shows activation as follows:

(2) $$\begin{array}{c} A\left(\theta_{j};S{}^{'},\sigma_{tuning}\right)=\frac{\sum_{i=1}^{n}a\left(\theta_{j};S{{}^{'}}_{i},\sigma_{tuning}\right)}{n},\; \end{array}$$

where *n* is the number of individual items presented in a stimulus.

For more biological plausibility, we added a random Poisson noise (e.g., as in Brezis et al., 2017; Jazayeri & Movshon, 2006) to each layer 2 neuron. That is, the spike frequency *Aʹ*(θ; *Sʹ*, σ_tuning_) of a neuron preferring orientation θ_j_ with a tuning function width of σ_tuning_ was randomly drawn from a Poisson distribution (*Pois*) with a mean of *A*(θ*_j_*; σ_tuning_) + 4, where 4 was added as an average default spike rate of each neuron (baseline noise):

(3) $$\begin{array}{c} \;\;\;\;\;\;A^{'}\left(\theta_{j};S^{'},\sigma_{\mathrm{tuning}}\right)∼Pois\left[A\left(\theta_{j};S^{'},\sigma_{\mathrm{tuning}}\right)+4\right]\; \end{array}$$

#### Decoding rules

**Figure 3.**
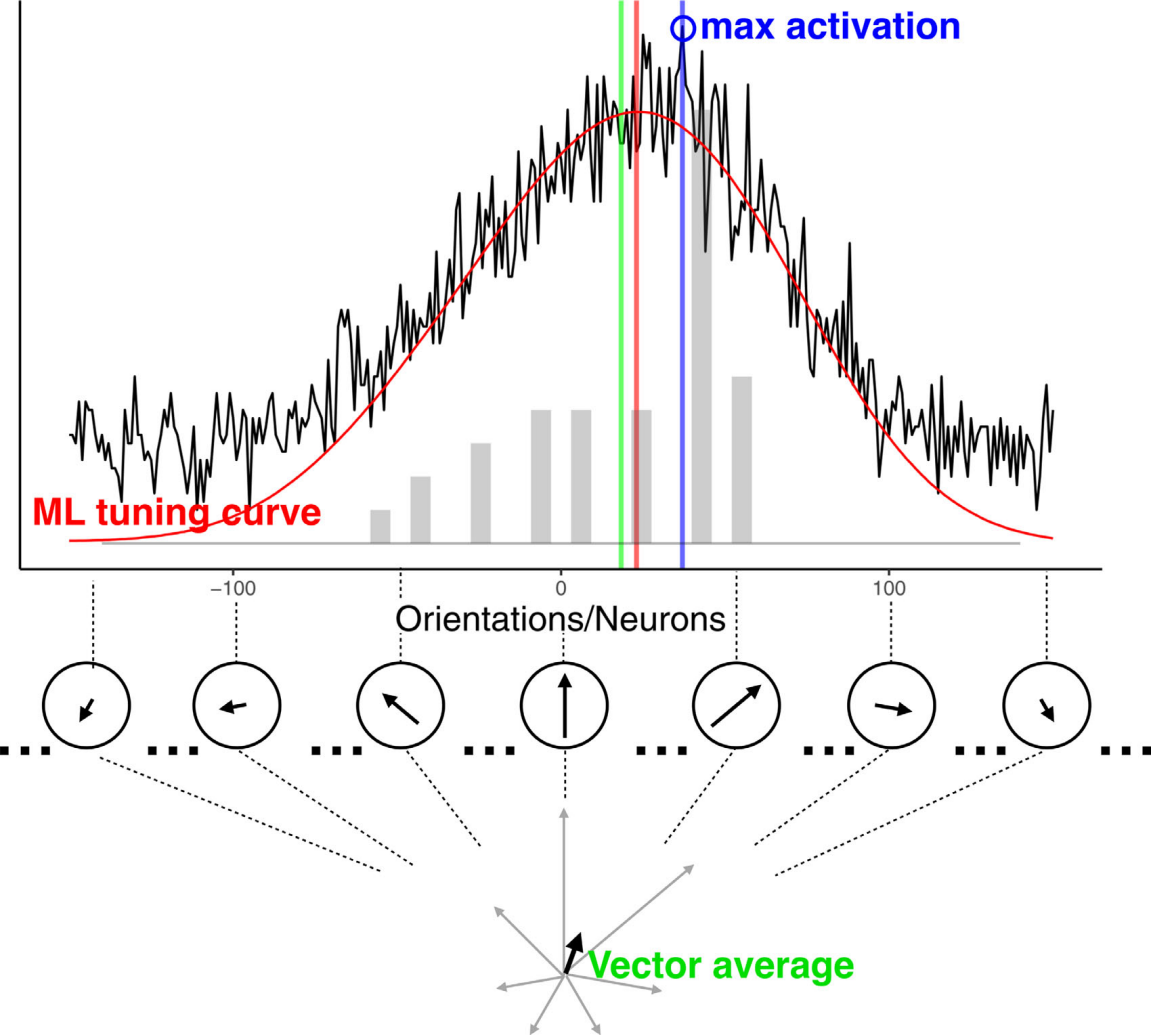
An illustration of different decoding rules applied to a noisy population response to an example orientation distribution (shown as the gray bars). The population response is corrupted by Poisson noise (solid black bumpy line) Decoded mean orientations as a function of the decoding rule are shown with colored vertical lines as follows: *PM_WTA_* (blue), *PM_MLE_* (red), and *PM_VA_* (green). The underlying estimators are shown accordingly. Note that the *y* axis is not labeled because it combines three different scales and units for the neuron response, the maximum likelihood estimation, and the stimulus distribution.

To decode the perceived mean from the population response, we applied three different rules often tested in the literature (Dakin et al., 2005; Webb et al., 2007, 2010): WTA, vector average (VA), and maximum likelihood estimation (MLE) (Figure 3). In the WTA rule, a neuron showing the maximum activation among *m* layer 2 neurons is found and its preference defined by the peak of its tuning curve was taken as the perceived mean (*PM*), so that:

(4) $$\begin{array}{c} PM_{WTA}=\underset{\theta }{argmax}\left[A{}^{'}\left(\theta ;S{}^{'},\sigma_{tuning}\right)\right].\; \end{array}$$

In the VA rule, the perceived mean is the direction of a resultant of individual vectors whose directions and length are defined by preferred orientations θ_j_ and activation (*A*’(θ*_j_*)) of each of the *m* layer 2 neurons:

(5) $$\begin{array}{ccc} & & PM_{VA}\\ & & =arctan\left(\frac{\sum_{j=1}^{m}A{}^{'}\left(\theta_{j};S{}^{'},\sigma_{tuning}\right)\times sin\theta_{j}}{\sum_{j=1}^{m}A{}^{'}\left(\theta_{j};S{}^{'},\sigma_{tuning}\right)\times cos\theta_{j}}\right).\; \end{array}$$

In the MLE rule, the perceived mean is an orientation θ*_k_* whose presentation would be most likely to cause the observed population response given the shape of the tuning curves (σ_tuning_), as defined by equation 1. The log-likelihood of the population response under each θ was determined by the Poisson density function (*f_Pois_*) applied to the spike rate *A*’(θ*_j_*) of each layer 2 neuron:

(6) $$\begin{array}{ccc} & & L\left(\theta ;\sigma_{tuning}\right)\\ & & =\sum_{j=1}^{m}logf_{Pois}\left[\theta ;A{}^{'}\left(\theta_{j};S{}^{'},\sigma_{tuning}\right)\right].\; \end{array}$$

Then, the perceived mean was defined as:

(7) $$\begin{array}{c} PM_{MLE}=\underset{\theta }{argmax}L\left(\theta ;\sigma_{tuning}\right).\; \end{array}$$

#### Simulations and fitting the population coding model to the data

To model a pooled population response, we created a vector of model neurons, each preferring a single orientation in a 360° circular space. The step between two closest neurons in preferences was 0.1°. Although the orientation space as a simple feature dimension spans across 180° (e.g., 200° equals 20° if the stimulus is an oriented bar), we used the 360° space, because we defined the average orientation as a mean apex direction of the triangles (200° is a different apex orientation than 20°).

We first predicted how big the bias from a real mean orientation should be as a function of the tuning curve width parameter (σ_tuning_) for each stimulus distribution from our experiment. The range of tuning width that we simulated for was from 20° to 90° with a step of 1°. For each stimulus mode-mean distance *d* ∈ {−20.0°, −13.7°, −7.0°, 0°, 7.0°, 13.7°, 20.0°}, each tuning width σ_tuning_ ∈ {20°, 21°, …, 90°}, and each decoding rule *r* ∈ {WTA, VA, MLE}, we ran 5,000 Monte Carlo simulations with a fixed σ_early_ parameter, as specified elsewhere in this article. The importance of including the early noise applied to any single item into the model is explained by the fact that it can systematically shift the peak location of an encoded distribution if the generative stimulus distribution is skewed. The mean output of the 5,000 simulations termed *m*(*d*, σ_tuning_, *r*) was taken as the model's predicted bias (from each of the decoding rules) for a given combination of *d*, σ_tuning_, and *r*. The SD of the simulation outputs termed σ(*d*, σ_tuning_, *r*) was taken as a measure of decoding noise for a given combination of *d*, σ_tuning_, and *r*.

Apart from the σ_tuning_ determining the bias and the decoding noise, our model included two more parameters to account for other sources of systematic and non-systematic response variability, constant error and late noise. The constant error reflects an overall clockwise or counterclockwise bias that observers could systematically show in all responses regardless of the feature distribution. In our model, it was defined as a constant added to all predicted average decoded means in all distributions, thus shifting them either to a positive (clockwise) or to a negative (counterclockwise) direction. The range of tested constant errors was from −3° to 3°, with steps of 0.1°. Late noise (σ_late_) is a parameter that accounts for response variability beyond the early input noise and the decoding noise in our model (e.g., memory distortions, decision and motor factors). The range of tested σ_late_ was from 0° to 40°, with steps of 1°.

We used a combination of MLE and a grid search algorithm to find the most likely combination of parameters σ_tuning_, σ_late_, constant error, and decision rule *r* for each observer. For each *k*-th trial completed by the observer, we estimated the parameter likelihood as the probability of an observed response error *E_k_* (as defined in Design and data analysis section) under a normal distribution with mean *m*(*d_k_*, σ_tuning_, *r*) + Constant error and a SD defined as a linear combination of variances of decoding noise and late noise*.* The log-likelihood for all trials, therefore, was defined as follows:

(8) LE;r,σtuning,CE,σlate=∑logfN12Ek;mdk,σtuning,r+CE,σ2dk,σtuning,r+σlate2.

#### Loss function model

To test how realistic prediction our population coding model does compared with one of the previous computational models of robust averaging, we also fit the loss function model from Teng et al. (2021). They found that an inverse Gaussian loss function was best to predict performance in their motion averaging task. Here, we implemented the same model (see Teng et al., 2021 for a detailed description). The core tested parameters of that model were the SD of the loss function (σ_loss_) and the size of a random sample of items (*N*) to which the loss function is applied. The tested σ_loss_ ranged from 1° to 50°, with steps of 1°. The tested *N* ranged from 2 to 24 or 36 (depending on the total number of items in a set), with steps of 1. For each combination of parameters, we ran 5,000 simulations. There were two modifications, however, as to what noise parameters we included in our simulations. Whereas Teng et al. (2021) did not include any early noise to their model, we did include it into our version of their model (σ_early_ = 4°) as a fixed parameter, which is important, in our opinion, for the reason explained elsewhere in this article. As in the population coding model, we also included the late noise and the constant error. The most likely parameters model was found with MLE the same way, as described for the population coding model, although the search has been made across four rather than three parameters.

#### Model estimation and comparison

We formally compared the four models (three versions of the population coding model with different decoding rules and the loss function model) using the Akaike information criterion (AIC). To estimate AIC we used log likelihood calculated using Equation (8). Overall, we followed the method used by Teng et al. (2021). We first calculated the ΔAIC of four models for each participant in a way that the smallest AIC across four models was subtracted from the AIC of each model. Then, the resulting ΔAICs were summed across participants for each model. These summed ΔAICs were used to compare the models, such that the smaller the summed ΔAIC, the better the model fits the data.

### Results and discussion

**Figure 4.**
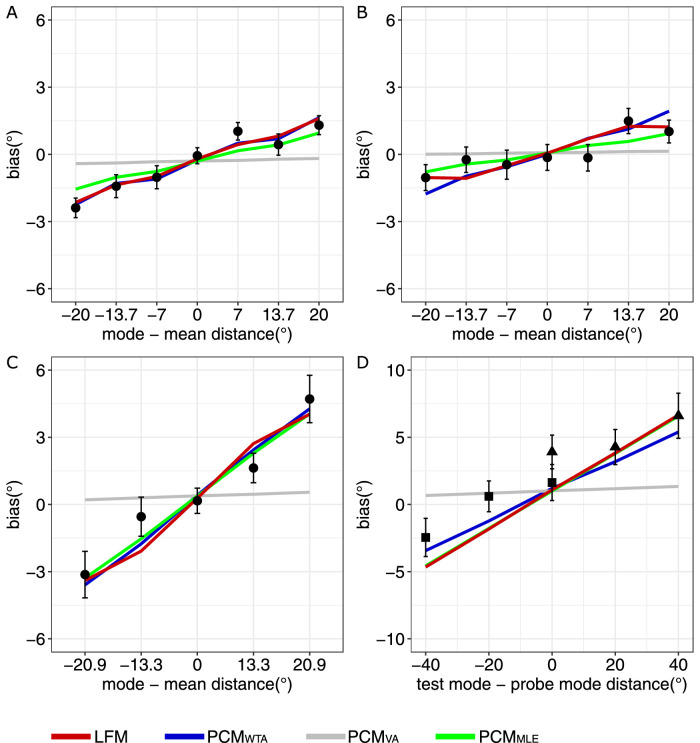
Results of Experiments 1–4. The plots show the adjustment bias as a function of mode–mean distance in (A) Experiment 1, (B) Experiment 2, (C) Experiment 3, or as a function of distance between the modes of a test set and a sample set in (D) Experiment 4. Data points depict average observed biases. Error bars show standard error of the mean with between-subject variance removed following the Cousineau (2005) method. The lines show the model predictions averaged across participants. Note that in (D), the biases in conditions with the right- (squares) and left-skewed (triangles) test distributions are shown with different data point shapes. As some models predict the same biases, blue and red lines on (A) and red and green lines on (D) substantially overlap.

**Figure 5.**
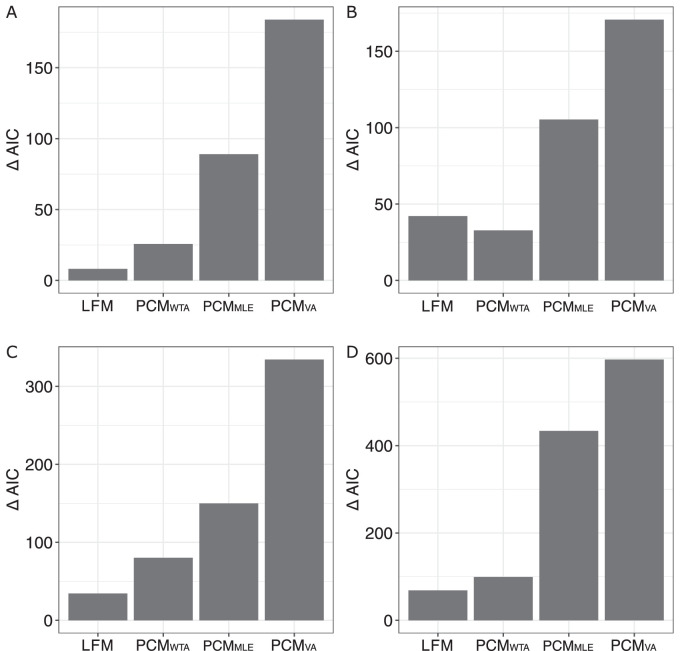
Results of Experiments 1–4. The plots show change in the Akaike information criterion (ΔAIC) summed across participants for four models in (A) Experiment 1, (B) Experiment 2, (C) Experiment 3, and (D) Experiment 4. The smaller the ΔAIC, the more likely the model was.

We observed systematic biases away from the real mean toward the mode of the orientation distributions that tended to grow in an absolute magnitude as a function of skewness (Figure 4A). The slope of the bias-skew function was positive (slope = 0.09) significantly greater than zero, t(11) = 5.04, *p* < 0.001. In addition to the bias caused by the skew of the distribution, there was a small intercept (−0.3° based on lmm best fit) that we interpret as a counterclockwise constant error. Both the population coding model and the loss function model showed very similar performance (Figure 4A). Model comparison showed that the loss function model showed the best overall fit of our data (ΔAIC = 8.18) (Figure 5A). The population coding model with the WTA decoding rule (*PCM_WTA_*) showed the best fit (*PCM_WTA_* ΔAIC = 25.73) among the three versions of the population coding models (Figure 5A). The participants’ median best fit parameters of the *PCM_WTA_* were as follows: σ_tuning_ = 35°, constant error = −0.1°, and σ_late_ = 6° (here and in other experiments, we provide the parameters only for the best version of the population coding model). The parameters of the loss function were as follows: σ_loss_ = 27.5°, N = 5, constant error = −0.05°, σ_late_ = 12°.

Overall, our results showed that observers tended to estimate the mean orientation of the skewed distributions as being biased toward the mode of these distributions. The greater the distance between the mode and the mean was, the greater the bias we observed. This finding is consistent with the results by Teng et al. (2021) for motion direction averaging. This bias cannot be explained merely by arithmetic averaging, because this mechanism predicts unbiased estimates. Potentially, this indicates unequal consideration of items from the peak and the long-tail parts of the distribution, in accordance with the idea of robust averaging. We found that both the population coding model (*PCM_WTA_*) and the loss function model could capture this pattern quite accurately, yet, the latter model was shown to be more preferable in terms of likelihood.

## Experiment 2

Because of the coronavirus disease 2019 pandemic restrictions on in-laboratory testing, we moved our further experiments to an online platform. Although online experiments are considered to be a proper alternative to offline psychophysics studies (e.g., Bridges, Pitiot, MacAskill, & Peirce, 2020; Chetverikov & Upravitelev, 2016; McGraw, Tew, & Williams, 2000; Semmelmann & Weigelt, 2017), the data collected online in inexperienced observers still can differ from the data acquired offline in psychophysically experienced observers. Keeping in mind possible differences in the observed effects owing to online testing, Experiment 2 was an online replication of Experiment 1 that we used as a reference for further online experiments described in this article.

### Participants

Fifty-one observers, 22 female, mean age of 25.7 ± 9.5 years, were recruited via Prolific (www.prolific.ac) for participation. They were paid £1.5 for participation in the experiment that lasted about 12 minutes on average. All participants reported having normal or corrected to normal vision and no neurological problems. All observers gave electronic informed consent before the experiment in accordance with the Declaration of Helsinki. The data of two participants and 71 individual trials (approximately 1%) were excluded from the analysis based on the exclusion criteria described in Experiment 1. Therefore, the data from 49 participants were analyzed.

### Stimuli, procedure, and design

The experiment was made using PsychoPy 3 (Peirce et al., 2019) and run online via Pavlovia platform (pavlovia.org). The experiment was available to run only on desktop computers or laptops, using a computer mouse or a touchpad for orientation adjustment. Stimuli were the same as in Experiment 1 in terms of the feature distributions, time course, responses, and so on. The only difference concerned the number of trials per data point. To make the experiment short and not exhausting for our online participants, the number of trials per condition was reduced to 20, compared with 100 in Experiment 1. The whole experimental session included 140 trials (7 skew conditions as in Experiment 1, 20 trials per condition). The potential loss in measurement precision associated with shortening the number of trials was compensated for by collecting data from more observers.

### Results and discussion

The linear mixed-model analysis showed the positive slope of the bias-skew function, slope = 0.05, t(48) = 2.65, *p* < 0.01, suggesting that the systematic bias toward the mode of the orientation distribution growing with the mode–mean distance (Figure 4B). Model comparison showed that the *PCM_WTA_* better fit the data, ΔAIC = 32.79, than the loss function model, ΔAIC = 42.12, and that the loss function was followed by the other two models (Figure 5B). The median best fit parameters of the two best models were as follows: for the *PCM_WTA_*, σ_tuning_ = 42°, constant error = 0.2°, and σ_late_ = 7°; for the loss function model, σ_loss_ = 20°, *N* = 4, constant error = 0.2°, and σ_late_ = 20°.

We conclude that the observed pattern of results in Experiment 2 was similar to that observed in Experiment 1. In particular, we again observed a bias toward the mode increasing with the mode–mean distance. Therefore, Experiment 2 succeeded in replicating the data from the in-laboratory Experiment 1. However, a model comparison yielded different results from Experiment 1 pointing to an advantage of the population coding model in terms of fit.

## Experiment 3

**Figure 6.**
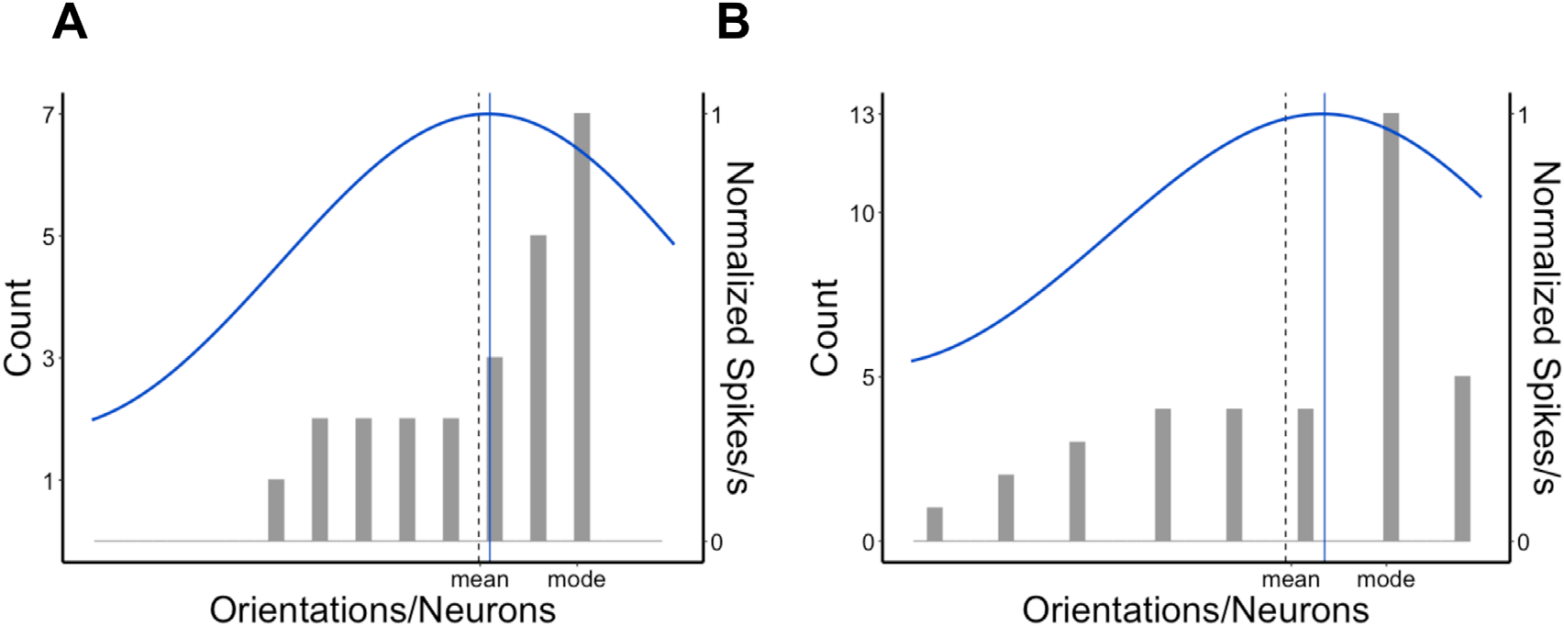
Two feature distributions (histograms) with similar mode–mean distance (≈20°) but different shapes and corresponding model population responses (blue curves) normalized by peak activation. The population responses are modeled for the σ_tuning_ = 40°. In (A), a distribution from Experiments 1 and 2 is shown; in (B) a distribution from Experiment 3 is shown. As can be seen, even though the mean–mode distance is the same, the peak population response (blue vertical line) is shifted away from the mean (dashed vertical line) much stronger in (B) than in (A).

In Teng et al. (2021) and in our Experiments 1 and 2, the direction and magnitude of a bias is tracked as a function of the mode–mean distance. However, the population coding model implies that the properties of the ensemble representation, including the location of a peak interpreted as an ensemble average, depends on the overall distribution of neural responses of a pooling population. This entire shape depends on the tuning properties of pooling neurons (σ_tuning_) and a feature distribution of a stimulus. In Experiment 3, we tested this prediction of the model by using new shapes of feature distributions while keeping the mode–mean distances almost the same as in Experiments 1 and 2. Specifically, we generated distributions in such a way that we might expect a greater bias away from the mean than in Experiments 1 and 2, given equal mode–mean distances. In Figure 6, we show an example of two such distributions (one from Experiment 1 and another from Experiment 3) with predicted population responses to these distributions. Note that these population responses are predicted based on tuning properties from an expected range based on our model fits in these experiments. Accordingly, we were also interested in how both the population coding model and the loss function model would perform in accounting for the data obtained from these new distributions.

### Participants

Fifty observers, 28 females, mean age 24.2 ± 5.5 years, were recruited via Prolific (www.prolific.ac) to take part in the experiment. They were paid £1.5 for participation in the experiment that lasted approximately 13 minutes. All observers reported having normal or corrected to normal vision and no neurological problems. All observers gave electronic informed consent before the experiment in accordance with the Declaration of Helsinki. The data of 4 participants and 73 individual trials (approximately 1.3%) were excluded from the analysis based on our exclusion criteria described in Experiment 1. Therefore, the data of 46 participants were analyzed.

### Stimuli, procedure, and design

The experiment was run online via Pavlovia platform, under the same restrictions on equipment as in Experiment 2. Thirty-six isosceles white oriented triangles were shown as a sample set in each trial. Each triangle had 13 pixels width and 26 pixels height. The stimuli were located on the 6 × 6 imaginary grid that occupied the central 480 × 480-pixel area of the screen. Each cell (80 × 80 pixels in size) could place one triangle placed at the cell center with a random jitter of 15 pixels along both axes.

**Figure 7.**
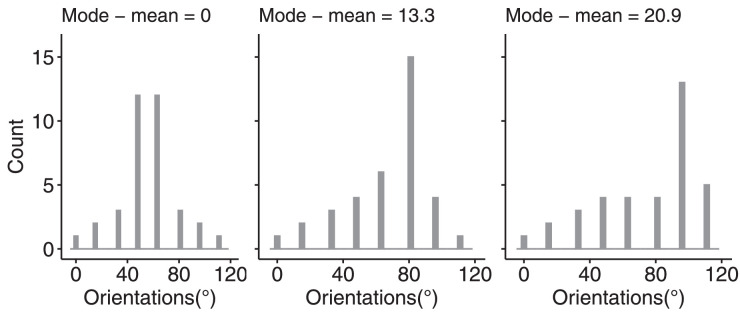
Orientation distributions used in Experiment 3. Only left-skewed distributions are shown in this example, whereas the whole design included the right-skewed distributions as well.

To generate orientation distributions, we used an approach similar to that from Teng et al. (2021), but with a modification. We generated mixtures of two Gaussian distributions, one with a SD of 7.5° and another with a SD of 30°. We varied the distance between the means of these two distributions to accomplish various mode–mean distances of the overall mixture distribution. We then discretized our mixed distributions to make them fixed-shaped within each condition and equally spaced, as in Experiments 1 and 2. The distributions covered a range of 112° with a step of 16°. The frequency of each discrete orientation *x*° was approximately proportional to the probability density within an interval [*x*° – 10°; *x*° + 10] of the generative mixture distribution. Using this method, we created distributions whose mode–mean distances were most similar to those from Experiments 1 and 2, namely, 0°, ±13.3°, and ±20.9°. For these distributions, our preliminary simulations predicted biases approximately three to four times as large in magnitude as in the corresponding distributions from Experiments 1 and 2, given that the tuning curves of model neurons are in the range of 40° to 45°. We did not use the distribution with the smallest mode–mean distance from Experiments 1 and 2 ( ±7°), because these distributions failed to produce a sufficient bias in Experiment 2 to make a reliable prediction. Overall, therefore, we had five fixed distributions with various amounts and directions of skewness. Histograms of these distributions are shown in Figure 7. The mean orientation of the set ensemble was randomly assigned for each trial in the same way as described in Experiment 1. Observers gave their responses using the adjustment method (also see Experiment 1 for description).

### Results and discussion

As in the two previous experiments, the linear mixed model showed a significant positive slope of the bias-skew function, slope = 0.16, t(45) = 4.54, *p* < 0.001, suggesting the bias toward the mode growing in magnitude as a function of mode–mean distance (Figure 4C). Importantly, this slope was also steeper compared with the slope observed in Experiment 2, 0.16 vs. 0.05, t(94) = 2.69, *p* < 0.01. The loss function model showed the best fit to the data, ΔAIC = 34.46 (Figure 5C). The *PCM_WTA_* was the best among the three versions of the population coding model, ΔAIC = 80.23 (Figure 5C). The best fit median parameters across participants of the best population coding model and the loss function model were as follows: for *PCM_WTA_*: σ_tuning_ = 51.5°, constant error = 0.6°, and σ_late_ = 14.5°; for the loss function model: σ_loss_ = 31.5°, N = 5, bias = 0.4°, and σ_late_ = 14°.

The main new finding from Experiment 3 was the larger slope of the bias-skew function compared with Experiment 2. This finding is basically consistent with our model predictions for this experiment. To remind, we changed the shapes of the distributions while keeping the mode–mean distance about the same as in Experiments 1 and 2. We conclude, therefore, that the ensemble representation is indeed more than just the mean or mode (or other kind of summary statistic) but the representation of the whole feature distribution (Chetverikiv, Campana, & Kristjansson, 2016, 2017b; Kim & Chong, 2020). This finding is strongly in line with the idea of pooled population response. At the same time, we can also see that the loss function model also fits the observed pattern well.

## Experiment 4

In Experiments 1 through 3, our observers saw sets of triangles as sample stimuli and adjusted their mean orientation using a single triangle. In Experiment 4, we changed the test stimulus from the single triangle to another set. If the response bias is indeed associated with the population representation of the whole distribution, then this bias should be eliminated if the sample and the test distributions have the same shape but it should increase if their shapes are different. For example, if both a sample and a test distributions are right skewed relative to the mean, the observer will adjust the test stimulus so that the population peaks match, but this will make the means match as well. So, there will be no observable bias in the data even though the perceived means are biased in both sets. However, if the sample set is right skewed and the test set is left skewed, we can expect a bias twice as big as we could observe with a single triangle or a symmetrically distributed feature distribution. Hence, Experiment 4, apart from testing the idea of distribution-dependent ensemble representations, is an important test for the so-called El Greco fallacy (Firestone & Scholl, 2016), which should show whether the biases we observed in the previous experiments are perceptual in nature.

### Participants

Fifty observers, 33 females, mean age 26.9 ± 9.5 years were recruited via Prolific (Prolific.co) to participate in the experiment. The observers were paid £1.5 for completing the experiment that lasted approximately 18 minutes. All observers reported having normal or corrected to normal vision and no neurological problems. All observers gave electronic informed consent before the experiment in accordance with the Declaration of Helsinki. The data of 5 observers and 63 individual trials (approximately 1.1%) were excluded from the data as their adjustment accuracy did not reach the requirements described in the data analysis section. Thus, the data of 45 participants were analyzed.

### Stimuli, procedure, and design

The experiment was run online via Pavlovia platform, under the same restrictions on equipment as in Experiments 2 and 3. Stimuli and procedure were similar to Experiment 3 except for some modifications to feature distributions and the adjustment procedure. First, we used only those feature distributions from Experiment 3 whose mode–mean distances were ±20.9° or 0°. The sample set could be either right-skewed, mode–mean distance = 20.9°; or left-skewed, mode–mean distance = –20.9°. The test set could be right skewed, left skewed, or symmetric, mean–mode distance = 0°. Observers were instructed to adjust the mean orientation of the test set to the mean orientation of the just seen sample set by clicking on or dragging the mouse cursor with the left button of a computer mouse around the center of the screen. The location of a mouse cursor on an imaginary circumference with a center at fixation defined the real circular mean orientation of the test set. Unlike Experiments 1 through 3, we did not present an orientation ring with a slider during adjustment. This was done to reduce a visible discrepancy between the perceived mean (which could be biased) and the real mean indicated by the slider location. Both sample and test sets mean sizes, as well as locations of individual triangles on a screen were independently randomized in the same way as test sets did in Experiment 3.

The skew of the sample and the test sets were manipulated orthogonally yielding 2 (sample set skew: 20.9° or −20.9°) × 3 (test set skew: 20.9°, 0° or −20.9°) = 6 combinations of conditions. We then used the distance between the mode of a test distribution and a sample distribution taken within the same range (e.g., both in range from 0° to 112°) as an independent variable. If the sample set was right skewed, then the test sample distance could be 0° (test right skewed), –20° (test symmetric), or –40° (test left skewed). If the sample set was left skewed, then the distance could be 0° (test left skewed), 20° (test symmetric), or 40° (test right skewed). Therefore, we analyzed the bias as a function of five test sample mode differences: −40°, −20°, 0°, 20°, and 40°.

In Experiment 4, we modified our criterion for data exclusion. We excluded participants whose overall error distribution across all conditions had a SD exceeding 80° (instead of 60°, as in the previous experiments). This change was explained by the fact that observers had to use a broadly spanned feature distribution to adjust the mean of another broadly spanned feature distribution. It is well-established in the literature that set discrimination becomes less precise when its variability increases (Dakin, 2001; Solomon, 2010), which is also the case for the adjustment method (Utochkin & Brady, 2020). So, our expectation is that the use of a set instead of a single item should contribute to the SD of the error distribution.

### Results and discussion

Our analysis using the linear mixed model showed that the bias tended to increase significantly as a function of test sample mode distance, slope = 0.11, t(44) = 3.94, *p* < 0.001. The intercept of the model was 2.43°, suggesting a considerable constant error. Most important, when the sample and the test distributions had opposite skew directions, there were strong biases in opposite directions (negative when the distance was –40° and positive when the test sample mode distance was 40°), but there were much less bias (except that associated with a constant error) when the sample and the test had the same direction of skew (Figure 4D).

Both the population coding and the loss function models performed well in accounting for these data. The loss function model was found to be slightly advantageous in terms of fit, ΔAIC = 68.59, compared with the *PCM_WTA_*, ΔAIC = 99.23, and they were then followed by other two versions of the population coding model (Figure 5D). The median best fit model parameters for the *PCM_WTA_* and the loss function models were as follows: for the *PCM_WTA_*, σ_tuning_ = 84°, constant error = 3°, and σ_late_ = 36°; for loss function model, σ_loss_ = 26°, *N* =3, constant error = 3°, and σ_late_ = 18°.

In summary, the bias grows as a function of distance between test and probe set modes. This growth indicates that the observers adjust the mean orientation by matching the peak population responses or minima of virtual loss function between the probe and the sample. This outcome goes in line with the findings from Experiments 1–3 with single items as tests.

## General discussion

Across four experiments, we found that the estimated average orientation of skewed distributions was systematically biased toward the mode and that the bias increased with the distance between the mode and mean orientation. This main finding is strongly consistent with the previously reported biases caused by asymmetrical feature distributions (Teng et al., 2021; Webb et al., 2007, 2010). Remarkably, we found that the biases were sensitive not only to the difference between the mode and the mean but to the entire shape of the distribution. As comparison between Experiments 2 and 3 shows, similar mean–mode distances could produce different magnitudes of bias if the whole distributions are different. This finding suggests that the perceived ensemble summaries are not completely determined by summary statistics of central tendency of the stimulus distribution. Rather, they are calculated from the population response whose shape correlates with but does not literally replicate the stimulus distribution. The importance of the whole feature distribution is consistent with recent evidence from other studies. For example, Kim and Chong (2020) demonstrated that observers were most accurate at mean size adjustment when the test stimulus was a set with a similar distribution with a sample in terms of set size, variance, and skewness. Experiments with perceptual priming also revealed the richness of ensemble representation. For instance, Michael, de Gardelle, and Summerfield (2014) showed that priming with a task-irrelevant ensemble speeds up the mean estimation of the task relevant ensemble when the variances of both ensembles matched. In another series of studies, Chetverikov, Campana, and Kristjánsson (2016, 2017a, 2017b, 2017c, 2019, 2020) used priming of the visual search to demonstrate that the whole shape of feature distribution that the observer is exposed to in several consecutive trials leaves its implicit trace in subsequent search, although conscious access is limited only to simple summary statistics, such as mean or variance (Hansmann-Roth, Kristjánsson, Whitney, & Chetverikov, 2021). Our main addition to this literature is evidence for the distribution-dependent changes in the perceived mean when the physical mean and mode remain the same.

Apart from just showing the skew-associated biases in orientation averaging, we tested various models of ensemble representation on our data. One model is the loss function model suggested by Teng et al. (2021) for the perception of skewed distributions that suggests a formal mathematical algorithm of evidence sampling and decision making based on this evidence. Another model is a neurally plausible population coding model (Utochkin et al., 2022) with three versions of decoding rules. These two kinds of models are not mutually exclusive but rather represent two different levels of models, in Marr's (1982) classification. The loss function model gives a good computational algorithm of sampling and application of a probabilistic weighting function to quantitatively predict the perceived average. The population coding model suggests more of the mechanistic implementation of any such computational algorithm. This model views averaging as a result of simple feedforward pooling of all presented features by a population of feature-selective neurons with large RFs (e.g., V4- or V5-like neurons) with a subsequent WTA decoding rule. Both kinds of models turned out to be quite accurate at predicting the data with their best fit parameters. Note that some of the previous works on central tendency perception have also considered formal and mechanistic population models as complementary (Li et al., 2017; Webb et al., 2007, 2010).

Although decoding operators were not the main target of our study, it can be useful to relate our results with the existing literature on that topic. Our experiments consistently showed that the WTA was the best decoding rule to account for the observed behavioral pattern. Although both WTA and MLE models predicted similar biases in skewed stimulus distributions, the WTA predicted greater bias magnitudes for the extremely skewed distribution. This emerges from the fact that the population response also becomes substantially skewed. In this case, Poisson noise further shifts the probability of getting the maximum response toward the shorter tail of the response distribution. In contrast, in MLE decoding, a symmetrical wrapped-normal tuning curve applied to a strongly skewed population response would shift the most likely preferred orientation toward the longer tail of the response distribution (i.e. closer to the stimulus mean orientation). There is a strong consensus among many researchers that maximum likelihood is the optimal rule for stimulus decoding from the population response (Dakin et al., 2005; Deneve, Latham, & Pouget, 1999; Jazayeri & Movshon, 2006; Seung & Somplolinski, 1993; Webb et al., 2007), because it takes into account the whole distribution of responses in the neural population and, hence, is more robust against effects of noise on individual neurons. In that sense, our data suggest a suboptimal decoding strategy implemented in behavior. In fact, this suboptimality is not surprising. The optimal character of maximum likelihood decoding is easy to demonstrate on model neural populations. However, there are examples showing that recovering the decoding rule from behavioral data leads to different conclusions depending on stimuli and the task (e.g., Nichols & Newsome, 2002; Webb et al., 2010). Our second important finding is that the VA rule is clearly the least plausible decoding mechanism, because it can hardly provide any sufficient bias to account for the data (Figure 4). An ability to strongly distinguish between the WTA and VA is an important advantage of using the skewed stimulus distributions, because they put the average preference and the peak preference on a substantial distance from each other.

Although both kinds of models considered in this article describe essentially the same computational process of decision-making based on multiple samples by the noisy observer, the population coding model makes an important addition to our understanding of how some fundamental properties of ensemble perception arise. Specifically, the previous formal computational models (de Gardelle & Summerfield, 2011; Li et al., 2017; Teng et al., 2021) do a perfect job at finding plausible functions that map individual feature values in a distribution to evidence weights for mean estimates and the ideal observer relying on these functions would show robust averaging. The population coding models (Baek & Chong, 2020a; Brezis et al., 2017; Dakin et al., 2005; Haberman & Whitney, 2012; Utochkin et al., 2022; Webb et al., 2007, 2010) suggest a parsimonious, biologically plausible explanation for how robust ensemble averaging can emerge from the combination of pooling information in the visual system and applying a specific decoding rule to this pooled population response. On one hand, it can be seen that the advantage of inlying elements over outlying elements typical for robust averaging is supported by the fact that neurons with feature preference in the middle of the stimulus distribution pool more signal than neurons with feature preference closer to the tails of that distribution. For example, even if the original feature distribution is uniform, its middle range will be over-represented in the population code. As Figure 1B shows, which neurons would have the strongest activation depends critically on their tuning properties. At the same time, robust averaging depends on the decoding rule applied to the pooled population response. As can be seen from our model predictions (Figure 4), some decision rules (such as VA) cause almost no bias in average estimates of the skewed distributions and as such they provide no robust averaging. In contrast, other decision rules, such as WTA or MLE, come closer to explaining biases in the real data.

One final remark should be made about the generality of the hypothetical mechanism presented in the population coding model. This model represents ensemble perception as a basically feedforward process of pooling local evidence by neural populations with large RFs and decision-making based on this pooled evidence. However, we do not deny a possibility that other sampling and computational strategies involving probabilistic updating based on feedback processes involving reweighting evidence from individual items can lead to robust averaging and outlier discount, though this role can be additional and these mechanisms take time to deploy (Epstein et al., 2020). Taking into account these feedback processes can be a potential direction for the further development of the existing population coding models of ensemble representation.
